# Dissemination of Cephalosporin Resistance Genes between *Escherichia coli* Strains from Farm Animals and Humans by Specific Plasmid Lineages

**DOI:** 10.1371/journal.pgen.1004776

**Published:** 2014-12-18

**Authors:** Mark de Been, Val F. Lanza, María de Toro, Jelle Scharringa, Wietske Dohmen, Yu Du, Juan Hu, Ying Lei, Ning Li, Ave Tooming-Klunderud, Dick J. J. Heederik, Ad C. Fluit, Marc J. M. Bonten, Rob J. L. Willems, Fernando de la Cruz, Willem van Schaik

**Affiliations:** 1 Department of Medical Microbiology, University Medical Center Utrecht, Utrecht, The Netherlands; 2 Instituto de Biomedicina y Biotecnología de Cantabria, Universidad de Cantabria-Sodercan-CSIC, Santander, Spain; 3 Institute for Risk Assessment Sciences, Division of Environmental Epidemiology, Utrecht University, Utrecht, The Netherlands; 4 BGI-Shenzhen, Shenzhen, China; 5 BGI-Europe, Copenhagen, Denmark; 6 Norwegian High-Throughput Sequencing Centre, Centre for Ecological and Evolutionary Synthesis, Department of Biosciences, University of Oslo, Oslo, Norway; MicroTrek Incorporated, United States of America

## Abstract

Third-generation cephalosporins are a class of β-lactam antibiotics that are often used for the treatment of human infections caused by Gram-negative bacteria, especially *Escherichia coli*. Worryingly, the incidence of human infections caused by third-generation cephalosporin-resistant *E. coli* is increasing worldwide. Recent studies have suggested that these *E. coli* strains, and their antibiotic resistance genes, can spread from food-producing animals, via the food-chain, to humans. However, these studies used traditional typing methods, which may not have provided sufficient resolution to reliably assess the relatedness of these strains. We therefore used whole-genome sequencing (WGS) to study the relatedness of cephalosporin-resistant *E. coli* from humans, chicken meat, poultry and pigs. One strain collection included pairs of human and poultry-associated strains that had previously been considered to be identical based on Multi-Locus Sequence Typing, plasmid typing and antibiotic resistance gene sequencing. The second collection included isolates from farmers and their pigs. WGS analysis revealed considerable heterogeneity between human and poultry-associated isolates. The most closely related pairs of strains from both sources carried 1263 Single-Nucleotide Polymorphisms (SNPs) per Mbp core genome. In contrast, epidemiologically linked strains from humans and pigs differed by only 1.8 SNPs per Mbp core genome. WGS-based plasmid reconstructions revealed three distinct plasmid lineages (IncI1- and IncK-type) that carried cephalosporin resistance genes of the Extended-Spectrum Beta-Lactamase (ESBL)- and AmpC-types. The plasmid backbones within each lineage were virtually identical and were shared by genetically unrelated human and animal isolates. Plasmid reconstructions from short-read sequencing data were validated by long-read DNA sequencing for two strains. Our findings failed to demonstrate evidence for recent clonal transmission of cephalosporin-resistant *E. coli* strains from poultry to humans, as has been suggested based on traditional, low-resolution typing methods. Instead, our data suggest that cephalosporin resistance genes are mainly disseminated in animals and humans via distinct plasmids.

## Author Summary

The rapid global rise of infections caused by *Escherichia coli* that are resistant to clinically relevant antimicrobials, including third-generation cephalosporins, is cause for concern. The intestinal tract of livestock, in particular poultry, is an important reservoir for drug resistant *E. coli*, but it is unknown to what extent these bacteria can spread to humans. Food is thought to be an important source because drug-resistant *E. coli* have been detected in animals raised for meat consumption and in meat products. Previous studies that used traditional, low-resolution, genetic typing methods found that drug resistant *E. coli* present in humans and poultry were indistinguishable from each other, suggesting dissemination of these bacteria through the food-chain to humans. However, by applying high-resolution, whole-genome sequencing methods, we did not find evidence for such transmission of bacteria through the food-chain. Instead, by employing a novel approach for the reconstruction of mobile genetic elements from whole-genome sequence data, we discovered that genetically unrelated *E. coli* isolates from both humans and animal sources carried nearly identical plasmids that encode third-generation cephalosporin resistance determinants. Our data suggest that cephalosporin resistance is mainly disseminated via the transfer of mobile genetic elements between animals and humans.

## Introduction

Antibiotic resistance among opportunistic pathogens is rapidly rising globally, hampering treatment of infections and increasing morbidity, mortality and health care costs [1], [2]. Of particular concern is the increased incidence of infections caused by *Escherichia coli* isolates producing extended-spectrum β-lactamases (ESBLs), which has rendered the use of third generation cephalosporins increasingly ineffective against this pathogen [3].

During the 1990s, the most commonly encountered ESBL genes were *bla*_TEM_ and *bla*_SHV_, and their spread occurred mainly through cross-transmission in hospitals. However, the epidemiology of ESBL-producing *E. coli* has changed. Nowadays, the most prevalent ESBL gene type is *bla*_CTX-M_ [4] and infections with ESBL-producing *E. coli* also occur in the community [5], [6]. The intestinal tracts of mammals and birds are important reservoirs for ESBL-producing *E. coli* [7], but it is unclear to what extent these bacteria can spread to humans. Food may be an important source, since ESBL genes have been detected in food-producing animals, especially poultry [8], [9], and on retail meat [10]. The presence of ESBL-producing bacteria in food has been attributed to widespread use of antimicrobials, including third generation cephalosporins, in industrial farming practices [11].

In The Netherlands, antibiotic use and prevalence of antibiotic resistance in humans are among the lowest in Europe [12], whereas antibiotic use in food-producing animals ranks among the highest in Europe [13]. These circumstances render The Netherlands particularly suitable to study the transfer of third-generation cephalosporin-resistant bacteria through the food-chain. Recent studies performed in The Netherlands suggested clonal transfer of ESBL-producing *E. coli* from poultry to humans [14]–[16]. However, these interpretations were based on typing methods that target a limited number of genes, and which may not have provided sufficient resolution to accurately monitor the epidemiology of pathogens [17]. In this study, we have therefore sequenced 28 ESBL-producing and four ESBL-negative *E. coli* strains that had previously been collected from humans, poultry, retail chicken meat and pigs and tested whether previous claims on the relationship between strains from different reservoirs could be confirmed at the whole-genome sequence level. Furthermore, we investigated the relatedness of cephalosporin resistance gene-carrying plasmids, which were derived from different backgrounds and reservoirs, at the genomic level.

## Results

### Sequencing of ESBL-producing *E. coli*

We assessed the relatedness of ESBL-producing *E. coli* from humans, animals and food by using Whole-Genome Sequencing (WGS). The genomes of 32, mostly ESBL-producing, *E. coli* strains isolated in The Netherlands in the period 2006–2011 were sequenced (Table 1). One set of isolates (n = 24) included five pairs of human and poultry-associated strains that had previously been found indistinguishable based on Multi Locus Sequence Typing (MLST), plasmid typing (pMLST) and ESBL gene sequencing [15], [18]. This set also included 11 human and poultry-associated isolates that carried an AmpC-type β-lactamase gene on an IncK plasmid [18]. The second set of isolates contained eight ESBL-producing strains that were isolated from pigs (n = 4) and their farmers (n = 4) (Table 1).

**Table 1:** *E. coli* strains sequenced in this study.

Strain	Source	Place of isolation	Date of isolation	MLST	ESBL	Inc-type of ESBL-carrying plasmid	AmpC	Inc-type of AmpC-carrying plasmid	GenBank BioProject
148	Human (blood)	Utrecht	14/02/2009	10	CTX-M-1	I1 (CC7, ST7)	n.d.	n.d.	PRJNA224190
320	Human (urine)	Utrecht	03/03/2009	10	TEM-52*	I1 (CC5, ST36)	n.d.	n.d.	PRJNA224195
681	Human (urine)	Delft	17/02/2009	10	TEM-52*	I1 (CC5, ST36)	n.d.	n.d.	PRJNA224196
38.27	Chicken (caecum)	Putten	14/06/2006	10	CTX-M-1	I1 (CC7, ST7)	n.d.	n.d.	PRJNA224199
38.34	Chicken (caecum)	Nunspeet^1^	20/06/2006	10	TEM-52*	I1 (CC5, ST10)	n.d.	n.d.	PRJNA224200
53A	Chicken meat	Utrecht^2^	10/05/2010	10	CTX-M-1	n.d.	n.d.	n.d.	PRJNA224201
85B	Chicken meat	Utrecht^3^	07/06/2010	10	TEM-52	n.d.	n.d.	n.d.	PRJNA224202
1240	Human (urine)	Schiedam	16/03/2009	58	CTX-M-1	n.d.	n.d.	n.d.	PRJNA224151
1350	Human (urine)	Leeuwarden	13/02/2009	58	CTX-M-1	I1 (CC7, ST7)	n.d.	n.d.	PRJNA224152
1365	Human (urine)	Leeuwarden	03/02/2009	58	CTX-M-1	I1 (CC7, ST7)	n.d.	n.d.	PRJNA224154
38.16	Chicken (caecum)	Nunspeet^1^	31/05/2006	58	CTX-M-1	I1 (CC7, ST7)	n.d.	n.d.	PRJNA224188
897	Human (pulmonary)	Terneuzen	22/02/2009	117	CTX-M-1	I1 (CC7, ST7)	n.d.	n.d.	PRJNA224139
1047	Human (faeces)	Velp	02/02/2009	117	CTX-M-1	I1 (CC7, ST7)	CMY-2	K	PRJNA224146
38.52	Chicken (caecum)	Nunspeet^1^	13/07/2006	117	CTX-M-1	I1 (CC7, ST7)	n.d.	n.d.	PRJNA224147
53C	Chicken meat	Utrecht^2^	10/05/2010	117	CTX-M-1	n.d.	CMY-2	K	PRJNA224234
435	Human (faeces)	Deventer	19/03/2009	68	CTX-M-1* †	n.d.	CMY-2‡	K	PRJNA224205
328	Human (urine)	Utrecht	04/03/2009	69	*negative*	n.d.	CMY-2‡	K	PRJNA224204
597	Human (urine)	Groningen	13/03/2009	95	*negative*	n.d.	CMY-2	K	PRJNA224228
668	Human (urine)	Delft	06/02/2009	648	CTX-M-15	n.d.	CMY-2‡	K	PRJNA224230
606	Human (pulmonary)	Groningen	18/02/2009	unknown	*negative*	n.d.	CMY-2‡	K	PRJNA224229
1A	Chicken meat	Utrecht^4^	2010	23	SHV-12	n.d.	CMY-2	K	PRJNA224231
27A	Chicken meat	Utrecht	26/04/2010	23	TEM-52	n.d.	CMY-2	K	PRJNA224233
9B	Chicken meat	Utrecht^4^	12/04/2010	93	SHV-12	n.d.	CMY-2	K	PRJNA224232
87A	Chicken meat	Utrecht^3^	07/06/2010	115	*negative*	n.d.	CMY-2	K	PRJNA224235
FAH1	Human (faeces)	farm A	18/04/2011	n.d.	CTX-M-1	n.d.	n.d.	n.d.	PRJNA224240
FAH2	Human (faeces)	farm A	19/04/2011	n.d.	CTX-M-1	n.d.	n.d.	n.d.	PRJNA224238
FAP1	Pig (faeces)	farm A	04/04/2011	n.d.	CTX-M-1	n.d.	n.d.	n.d.	PRJNA224241
FAP2	Pig (faeces)	farm A	04/04/2011	n.d.	CTX-M-1	n.d.	n.d.	n.d.	PRJNA224242
FBH1	Human (faeces)	farm B	24/05/2011	n.d.	CTX-M-1	n.d.	n.d.	n.d.	PRJNA224243
FBP1	Pig (faeces)	farm B	2011	n.d.	CTX-M-1	n.d.	n.d.	n.d.	PRJNA224244
FCH1	Human (faeces)	farm C	28/06/2011	n.d.	CTX-M-1	n.d.	n.d.	n.d.	PRJNA224245
FCP1	Pig (faeces)	farm C	28/06/2011	n.d.	CTX-M-1	n.d.	n.d.	n.d.	PRJNA224246

For all strains that were ESBL- and IncI1 plasmid-positive or AmpC- and IncK plasmid-positive, the Inc group of the plasmid that carried the ESBL/AmpC gene had previously been determined using a transformation-based approach [15], [18]. Identical numbers behind places of isolation indicate the exact same locations: i.e. the same retail store for chicken meat isolates, the same slaughterhouse for chicken isolates and the same farm (referred to as farms A, B, and C) for pig and pig farmer isolates.

* These ESBL genes were not found in the genome assemblies: *bla*_TEM-20_ or *bla*_TEM-1_ were found instead of *bla*_TEM-52_.

† This ESBL gene had previously only been typed using microarrays (no sequencing). The gene was found to belong to the CTX-M-1 group.

‡ These CMY genes were divided over two contigs that were connected on a scaffold. BLAST runs of the partial CMY-2 sequences against GenBank's nr database gave best hits with *bla*_CMY-2_ and mapping of raw Illumina reads against *bla*_CMY-2_ indicated that the full *bla*_CMY-2_ gene was present in the corresponding strain.

n.d.: not determined.

Illumina sequencing yielded draft genomes with an average assembly size of 5.2 Mbp (±0.17 Mbp), consisting of an average number of 133 scaffolds (±41) of size ≥500 bp and a mean N50 of 153 kbp (±47.9 kbp) (S1 Table). WGS-based MLST and ESBL gene analysis provided good agreement with previous typing data. Previously obtained MLST profiles and WGS-based MLST profiles were in complete agreement with each other. Although ESBL genes had previously been detected by both microarray-based methods and Sanger sequencing [15], the previously typed ESBL genes of four (out of 28) strains were absent from their assembled genomes. In three of these cases (strains 681, 320 and 38.34), we detected a *bla*_TEM-1_ or *bla*_TEM-20_ gene in the assembled genome, whereas a *bla*_TEM-52_ gene should have been found according to the typing data. Mapping the Illumina reads of these strains against their own assemblies showed that the assembled *bla*_TEM_ genes contained several ambiguous positions pointing to the presence of more than one type of *bla*_TEM_ gene (most likely a combination of *bla*_TEM-1_ and *bla*_TEM-52_) in these strains (S2 Table). In comparison, no ambiguous positions were found in the assembled *bla*_TEM_ genes of other strains using the same mapping approach. In addition, the relative coverage of the assembled *bla*_TEM_ genes of strains 681, 320 and 38.34 was higher than that of the assembled *bla*_TEM_ genes of other strains (S2 Table). These findings suggested that strains 681, 320 and 38.34 contain multiple nearly identical *bla*_TEM_ genes (i.e. *bla*_TEM-1_ and *bla*_TEM-52_) that hampered the correct assembly of these genes. The fourth inconsistency between WGS and typing data was the absence of *bla*_CTX-M-1_ from the assembly of strain 435. Mapping the reads of strain 435 against the *bla*_CTX-M-1_ gene sequence did suggest the presence of this gene in the WGS data, but with a depth of around 1/10^th^ the average genomic sequencing depth. Possible explanations include a relatively poor isolation efficiency of the *bla*_CTX-M-1_-carrying plasmid and/or the loss of this plasmid from the bacterial cells during culturing in the absence of antibiotics. The previous AmpC typing data [18] and our WGS data were in complete agreement.

### Phylogeny and epidemiology of ESBL-producing *E. coli*

**Figure 1:**
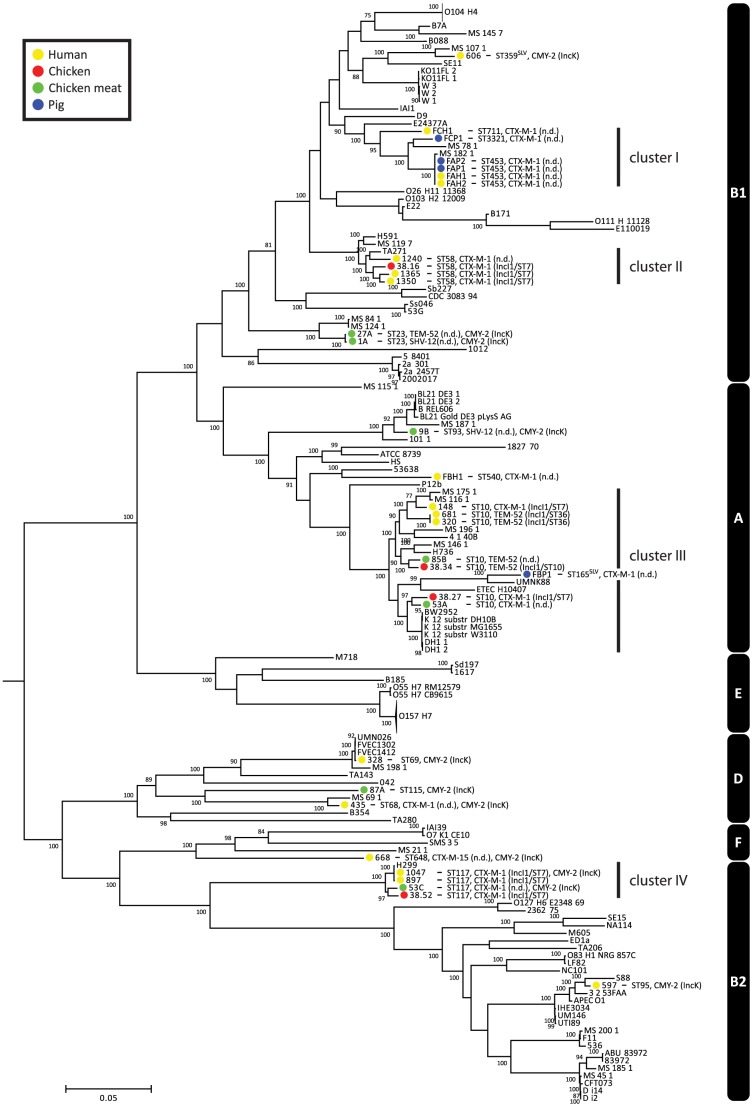
Phylogeny of *Escherichia* and *Shigella* species, including ESBL- and AmpC-positive strains sequenced for the purpose of this study. The tree was built using 18169 variable positions present in 215 core genes. The strains sequenced in this study are indicated in coloured bullets according to isolation source. Typing characteristics (Table 1) are given behind strain names. In case the MLST had not been determined before, it was determined using MLST v1.6 [55]. Clusters I–IV (see main text) are indicated behind the tree. Phylogroups are also indicated behind the tree (white text on black bars). The O104:H4 and O157:H7 branches are collapsed and represent 11 and 20 strains, respectively. Bootstrap support was implemented by running 100 bootstrap replicates. Values <75% are not displayed. SLV indicates Single Locus Variants of corresponding MLSTs.

To assess the phylogenetic context of the sequenced strains within the genus *Escherichia* and *Shigella*, we used publicly available genome sequences of *Escherichia* (n = 126) and *Shigella* (n = 12) strains. Based on COG assignments, we identified 215 core proteins in the 170 analysed genomes, from which a concatenated core genome alignment of 170461 bp was built. A phylogenetic tree based on the 18169 variable positions in this alignment confirmed previous clustering based around phylogroups A, B1, B2, D, E and F (Fig. 1) [19]. The sequenced strains clustered together in accordance with their ST. Strains did not cluster based on isolation source, year, plasmid or ESBL gene. The ESBL-producing strains were spread throughout the tree, indicating that acquisition of ESBLs arises in different *E. coli* genetic backgrounds and has occurred multiple times during evolution (Fig. 1).

There were four clusters of ESBL-producing strains isolated from humans and animals/meat (clusters I–IV, Fig. 1). Cluster I contained human and pig isolates from two pig farms, with strains from farm A being particularly closely related. The other three clusters contained the five pairs of human and chicken isolates that had previously been considered indistinguishable based on traditional typing methods [15].

**Figure 2:**
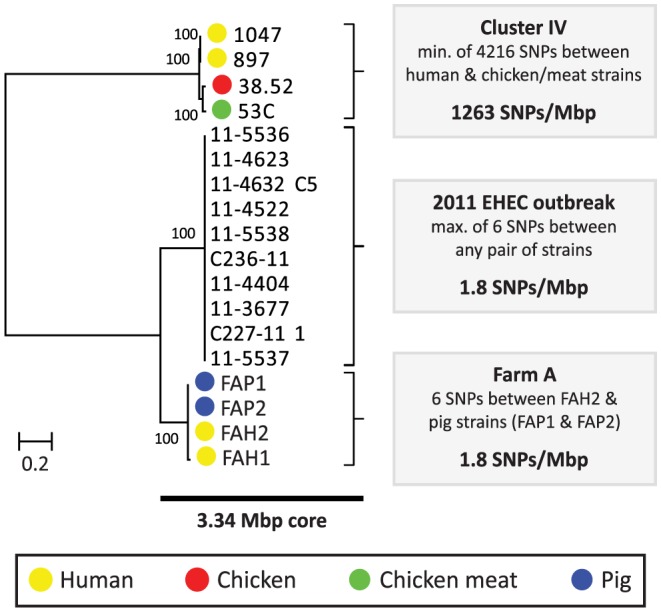
Phylogeny and SNP analysis of closely related ESBL-producing *E. coli* strains from human and poultry. A high resolution core genome analysis was performed for a subset of strains which, based on an initial phylogenetic analysis (Cluster IV, Fig. 1), included the most closely related pairs of human and poultry-associated ESBL-producing strains within our dataset. Strains within Cluster IV had previously been found to be identical with respect to MLST, ESBL gene and ESBL-carrying plasmid (Table 1). For comparative purposes, clonally related *E. coli* strains from the 2011 German EHEC outbreak [21] and four potentially clonally related strains isolated from a single pig farm (Farm A) were included in this analysis. A phylogenetic tree, built from the 107919 variable positions present in the resulting 3.34 Mbp core genome alignment is shown to the left. Bootstrap support was implemented by running 1000 bootstrap replicates. Coloured bullets refer to the isolation source. The number of SNPs found in each of the three clusters (Cluster IV, EHEC outbreak, Farm A) is shown to the right.

Among the five pairs of human and chicken isolates, the most closely related pairs were in cluster IV. The COG-based core genome alignment showed 171 SNPs between these strains, corresponding to 1003 SNPs/Mbp. To better elucidate the minimum number of SNPs between human and chicken isolates, we performed a core genome analysis using OrthoMCL [20] on the strains in cluster IV. For comparison, ten clonal O104:H4 strains from the 2011 German EHEC outbreak [21] and the four strains from pig farm A (cluster I) were included in this analysis (Fig. 1). We identified 3574 core proteins in this dataset translating to a concatenated nucleotide alignment of 3.34 Mbp. Within cluster IV there were 4216 SNPs between the most closely related isolates, corresponding to 1263 SNPs/Mbp. In contrast, only 0–6 SNPs (0–1.8 SNPs/Mbp) were found between any two strains in the German EHEC outbreak and only 6 SNPs were found between farmer isolate FAH2 and any of its two related pig isolates, suggesting recent clonal transmission of *E. coli* between pig and human in farm A (Fig. 2).

Given an estimated *E. coli* mutation rate of 2.3×10^−7^ to 3.0×10^−6^ substitutions per site per year [21], [22] and an average *E. coli* genome size of 5.2 Mbp, the number of SNPs (1263/Mbp) between the two most closely related human and chicken isolates largely exceeded the number of 3–41 SNPs that is expected to arise in 2.6 years (the difference in isolation dates between both strains, Table 1). Even if 10% of the detected SNPs were due to recombination, which is considerably more than the reported upper limit for recombinant DNA (∼3.5%) in *E. coli* [19], the number of SNPs due to mutation would exceed the expected maximum number of SNPs in case of recent clonal transmission. As the genetic distance between all other pairs of human and poultry isolates was even larger, our findings do not support a scenario of recent clonal transmission of ESBL-producing *E. coli* strains between humans and poultry.

### Reconstruction of plasmids from WGS data

**Figure 3:**
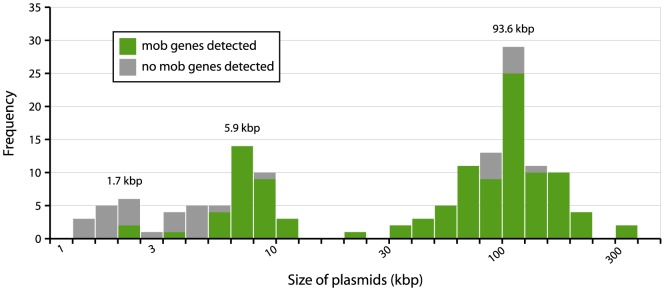
Distribution of plasmid sizes in the collection of 32 sequenced *E. coli* strains. The histogram shows the total number of reconstructed plasmids corresponding to each size class (in a logarithmic scale). The plasmid size distribution shows a trimodal abundance curve. Numbers above the three peaks refer to the median size for each class. Plasmids in which a relaxase gene was detected are shown in green and those in which it was not detected are shown in grey.

**Figure 4:**
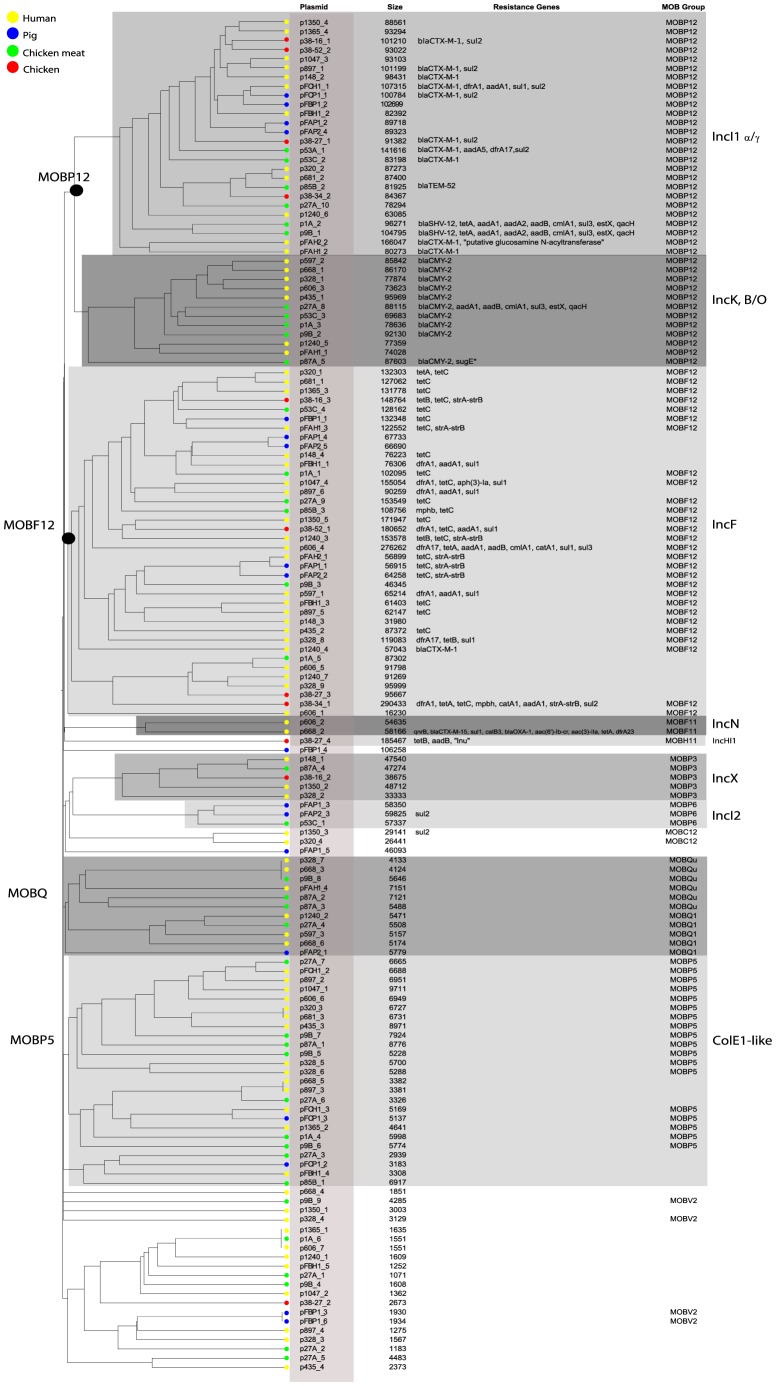
Hierarchical clustering dendrogram of reconstructed plasmids contained in the collection of 32 sequenced *E. coli* strains. The dendrogram was constructed as explained in Methods. Reconstructed plasmids are indicated with colored bullets according to isolation source. The dendrogram construction automatically grouped plasmids into Inc families, which are shown by background colours. Mob types are also indicated. Additional columns show plasmid sizes, resistance genes and MOB subfamilies.

To investigate the possibility of horizontal spread of ESBLs via plasmids, we employed a Plasmid Constellation Networks (PLACNET) approach to reconstruct plasmids from WGS data [23]. Application of this approach resulted in the reconstruction of 147 plasmids (average of 4.6±2.1 plasmids per strain), with plasmid sizes ranging from 1.1 kbp to 290.4 kbp (Table 2). The plasmid sizes showed a trimodal distribution (Fig. 3) that was similar to the distribution previously reported for plasmids from a wide range of bacterial taxa [24]. The median size of large (conjugative) plasmids was 93.6 kbp (n = 91). Small plasmids could be further subdivided into two groups: one with a median size of 5.9 kbp (n = 41), predominated by mobilizable plasmids (i.e. containing MOB genes) and one with a median size of 1.7 kbp (n = 15), predominated by non-mobilizable plasmids. Based on the classification of their MOB genes [25] and using a hierarchical clustering analysis of gene content (Fig. 4), reconstructed plasmids belonged to a limited number of plasmid families, of which the most abundant ones were IncF-MOB_F12_ (n = 38; average size of 107.4±57.7 kbp) and IncI1-MOB_P12_ (n = 26; average size of 95.7±20.0 kbp). Other abundant families included MOBP5 (n = 25), IncK (n = 12) and MOBQ (n = 11). Finally, there were 18, mostly small-sized, plasmids (median size of 1.6 kbp; range of 1.1–106.3 kbp) that were scattered throughout the dendrogram and could not be clearly subdivided into any family. A comparison between previous typing data and the PLACNET reconstructions showed that both data types were in excellent agreement with each other. First of all, the 11 strains that were previously found to contain an IncI1 plasmid were also found to contain such a plasmid using PLACNET. The sizes of these 11 reconstructed plasmids (average size of 92.7 kbp±5.7 kbp) were also in agreement with their previously estimated sizes on the basis of gel electrophoresis (average size of 97.7 kbp±3.8 kbp) [15]. Furthermore, the reconstructed plasmids for ten of these 11 strains had exactly the same ST as was previously found using pMLST. The only inconsistency was found for strain 38.34, which should contain an IncI1/ST10 plasmid according to pMLST, whereas we reconstructed an IncI1/ST36 plasmid. However, IncI1/ST10 and IncI1/ST36 are single locus variants that differ by only one SNP (http://pubmlst.org/plasmid/), indicating that this inconsistency was not a result of PLACNET, but was likely due to typing errors. Of the 11 strains that had previously been found to contain an IncK plasmid, ten were also found to contain such a plasmid using PLACNET, the only exception being strain 1047.

**Table 2:** Plasmids reconstructed using PLACNET.

Strain	IncI1	IncK	IncF	IncN	IncHI1	IncX	IncI2	MobC12	MobQu	MobQ1	MobP5	MobV2	Other	Nr. of plasmids	Total plasmid seq. (kbp) and scaffolds
148	p2: 98 (7), CTX-M-1	-	p3: 32 (2)	-	-	p1: 48 (3)	-	-	-	-	-	-	-	4	254 (18)
			p4: 76 (6)												
320	p2: 87 (5)*	-	p1: 132 (7)	-	-	-	-	p4: 26 (1)	-	-	p3: 7 (1)	-	-	4	253 (14)
681	p2: 87 (4)*	-	p1: 127 (4)	-	-	-	-	-	-	-	p3: 7 (1)	-	-	3	221 (9)
38.27	p1: 91 (8), CTX-M-1	-	p3: 96 (1)	-	p4: 185 (7)	-	-	-	-	-	-	-	p2: 3 (3)	4	375 (19)
38.34	p2: 84 (6)*	-	p1: 290 (41)	-	-	-	-	-	-	-	-	-	-	2	375 (47)
53A	p1: 142 (9), CTX-M-1	-	-	-	-	-	-	-	-	-	-	-	-	1	142 (9)
85B	p2: 82 (4), TEM-52	-	p3: 109 (14)	-	-	-	-	-	-	-	p1: 7 (1)	-	-	3	198 (19)
1240	p6: 63 (7)	p5: 77 (6)	p3: 154 (24)	-	-	-	-	-	-	p2: 5 (1)	-	-	p1: 2 (1)	7	449 (58)
			p4: 57 (18), CTX-M-1												
			p7: 91 (1)												
1350	p4: 89 (6)*	-	p5: 172 (47)	-	-	p2: 49 (1)	-	p3: 29 (5)	-	-	-	-	p1: 3 (1)	5	341 (60)
1365	p4: 93 (5)*	-	p3: 132 (7)	-	-	-	-	-	-	-	p2: 5 (1)	-	p1: 2 (1)	4	231 (14)
38.16	p1: 101 (7), CTX-M-1	-	p3: 149 (13)	-	-	p2: 39 (1)	-	-	-	-	-	-	-	3	289 (21)
897	p1: 101 (9), CTX-M-1	-	p5: 62 (3)	-	-	-	-	-	-	-	p2: 7 (1)	-	p4: 1 (2)	6	265 (23)
			p6: 90 (7)								p3: 3 (1)				
1047	p3: 93 (7)*	-*	p4: 155 (12)	-	-	-	-	-	-	-	p1: 10 (2)	-	p2: 1 (1)	4	259 (22)
38.52	p2: 93 (11)*	-	p1: 181 (40)	-	-	-	-	-	-	-	-	-	-	2	274 (51)
53C	p2: 83 (8), CTX-M-1	p3: 70 (5), CMY-2	p4: 128 (10)	-	-	-	p1: 57 (8)	-	-	-	-	-	-	4	338 (31)
435	-	p1: 96 (20), CMY-2	p2: 87 (2)	-	-	-	-	-	-	-	p3: 9 (1)	-	p4: 2 (1)	4	195 (24)
328	-	p1: 78 (7), CMY-2	p8: 119 (12)	-	-	p2: 33 (3)	-	-	p7: 4 (1)	-	p5: 6 (1)	p4: 3 (1)	p3: 2 (1)	9	346 (28)
			p9: 96 (1)								p6: 5 (1)				
597	-	p2: 86 (5), CMY-2	p1: 65 (5)	-	-	-	-	-	-	p3: 5 (1)	-	-	-	3	156 (11)
668	-	p1: 86 (9), CMY-2	-	p2: 58 (14), CTX-M-15	-	-	-	-	p3: 4 (1)	p6: 5 (1)	p5: 3 (1)	-	p4: 2 (1)	6	159 (27)
606	-	p3: 74 (7), CMY-2	p1: 16 (3)	p2: 55 (6)	-	-	-	-	-	-	p6: 7 (1)	-	p7: 2 (1)	7	521 (58)
			p4: 276 (36)												
			p5: 92 (4)												
1A	p2: 96 (10), SHV-12	p3: 79 (6), CMY-2	p1: 102 (7)	-	-	-	-	-	-	-	p4: 6 (1)	-	p6: 2 (1)	6	372 (26)
			p5: 87 (1)												
27A	p10: 78 (11)	p8: 88 (10), CMY-2	p9: 154 (20)	-	-	-	-	-	-	p4: 6 (1)	p3: 3 (1)	-	p1: 1 (1)	10	345 (48)
											p6: 3 (1)		p2: 1 (1)		
											p7: 7 (1)		p5: 4 (1)		
9B	p1: 105 (9), SHV	p2: 92 (13), CMY-2	p3: 46 (1)	-	-	-	-	-	p8: 6 (1)	-	p5: 5 (1)	p9: 4 (2)	p4: 2 (1)	9	274 (30)
											p6: 6 (1)				
											p7: 8 (1)				
87A	-	p5: 88 (29), CMY-2	-	-	-	p4: 47 (1)	-	-	p2: 7 (1)						
p3: 5 (1)	-	p1: 9 (1)	-	-	5	156 (33)									
FAH1	p2: 80 (20), CTX-M-1	p1: 74 (5)	p3: 123 (11)	-	-	-	-	-	p4: 7 (1)	-	-	-	-	4	284 (37)
FAH2	p2: 166 (20), CTX-M-1	-	p1: 57 (1)	-	-	-	-	-	-	-	-	-	-	2	223 (21)
FAP1	p2: 90 (6)	-	p1: 57 (1)	-	-	-	p3: 58 (7)	-	-	-	-	-	p5: 46 (2)	5	319 (26)
			p4: 68 (10)												
FAP2	p4: 89 (5)	-	p2: 64 (8)	-	-	-	p3: 60 (7)	-	-	p1: 6 (1)	-	-	-	5	286 (32)
			p5: 67 (11)												
FBH1	p2: 82 (8)	-	p1: 76 (9)	-	-	-	-	-	-	-	p4: 3 (1)	-	p5: 1 (1)	5	225 (20)
			p3: 61 (1)												
FBP1	p2: 103 (12)	-	p1: 132 (8)	-	-	-	-	-	-	-	-	p3: 2 (2)	p4: 106 (1)	5	345 (25)
												p6: 2 (2)			
FCH1	p1: 107 (10), CTX-M-1	-	-	-	-	-	-	-	-	-	p2: 7 (1)	-	-	3	119 (12)
											p3: 5 (1)				
FCP1	p1: 101 (10), CTX-M-1	-	-	-	-	-	-	-	-	-	p2: 3 (1)	-	-	3	109 (12)
											p3: 5 (1)				

**Figure 5:**
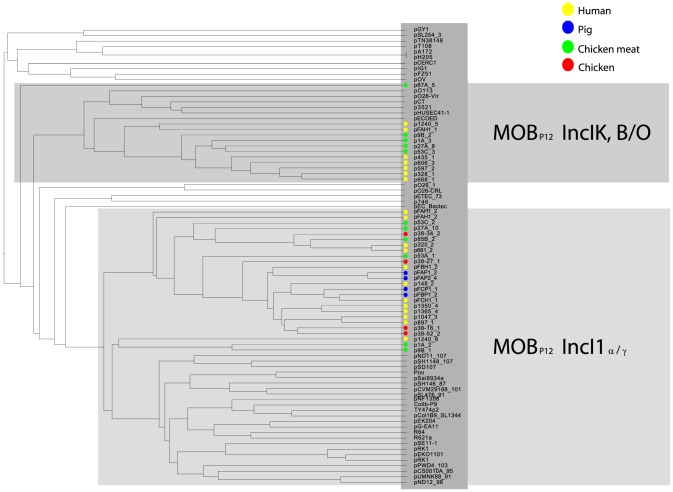
Hierarchical clustering dendrogram of reconstructed IncI1 and IncK plasmids contained in the collection of 32 sequenced *E. coli* strains together with relevant and similar reference plasmids. The dendrogram was constructed as explained in Methods. Reconstructed plasmids are indicated with colored bullets according to isolation source. All other (reference) plasmids were taken from public sequence repositories.

**Figure 6:**
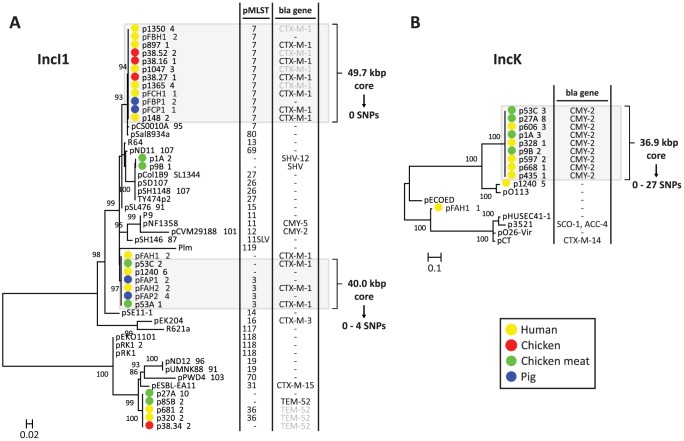
Phylogeny of reconstructed IncI1 and IncK plasmids and their closest relatives. Phylogenetic tree of IncI1 plasmids built from 763 variable positions present in an 8.6 kbp alignment, representing 8 core proteins (S3 Table) (A). Phylogenetic tree of IncK plasmids built from 2724 variable positions present in a 19.9 kbp alignment, representing 27 core proteins (S3 Table) (B). Bootstrap support was implemented by running 1000 bootstrap replicates. Reconstructed plasmids are indicated with coloured bullets according to isolation source (plasmid names include associated strain names, followed by a unique plasmid identifier). All other plasmids were taken from public sequence repositories. Plasmid STs (for IncI1 only) and encoded β-lactamases (with the exception of TEM-1, which does not provide resistance to third generation cephalosporins) are indicated to the right of the trees. 11SLV indicates a single locus variant of ST11. pMLST negative means that the reconstructed plasmid lacks one or more pMLST loci. *Bla* genes in light grey were not connected to the reconstructed plasmid, but should be located on this plasmid according to typing data. Light grey panels indicate potential epidemic *bla*_CTX-M-1_- and *bla*_CMY-2_-carrying plasmids. Core genome analysis of these plasmid subsets revealed virtually identical backbones of up to 50 kbp.

For each strain, the type and number of reconstructed plasmids are indicated in columns 2–14. Plasmid numbering (e.g. p1) corresponds with the numbering in Figs. 4–6, and is followed by the plasmid size (in kbp), the number of assigned scaffolds (between brackets), and assigned *bla*_ESBL_ or *bla*_CMY-2_ genes, if applicable. The same format is used for the last summarising column.

* Inconsistency between plasmid typing data and WGS data/PLACNET (typing data is between brackets below): 320 (*bla*_TEM-52_ on IncI1): *bla*_TEM-52_ not found in assembly and thus not linked to an IncI1 plasmid; 681 (*bla*_TEM-52_ on IncI1): *bla*_TEM-52_ not found in assembly and thus not linked to an IncI1 plasmid; 38.34 (*bla*_TEM-52_ on IncI1): *bla*_TEM-52_ not found in assembly and thus not linked to an IncI1 plasmid; 1350 (*bla*_CTX-M-1_ on IncI1): *bla*_CTX-M-1_ found in assembly, but not linked to an IncI1 plasmid; 1365 (*bla*_CTX-M-1_ on IncI1): *bla*_CTX-M-1_ found in assembly, but not linked to an IncI1 plasmid; 1047 (*bla*_CTX-M-1_ on IncI1): *bla*_CTX-M-1_ found in assembly, but not linked to an IncI1 plasmid; 1047 (*bla*_CMY-2_ on IncK): *bla*_CMY-2_ found in assembly, but no IncK plasmid reconstructed; 38.52 (*bla*_CTX-M-1_ on IncI1): *bla*_CTX-M-1_ found in assembly, but not linked to an IncI1 plasmid.

We also examined to what extent we were able to correctly connect ESBL and AmpC genes to reconstructed plasmids. Of the 28 previously typed ESBL genes, 24 were correctly identified in their genomes (Table 1) and among these, 15 were connected to a reconstructed plasmid. Four of the remaining nine unconnected ESBL genes (*bla*_CTX-M-1_ in strains 1350, 1365, 1047 and 38.52) should have been connected to an IncI1 plasmid according to previous typing data (Tables 1–2). The reason that these ESBL genes remained unassigned was because they were located on relatively small scaffolds (average size of 6.6 kbp) that did not contain enough genetic information to unequivocally match them to a single plasmid using our reference database. For the 15 cases where we were able to connect an ESBL gene to a reconstructed plasmid, typing data indicating where the ESBL gene should be located was available for four cases (strains 148, 897, 38.16 and 38.27) and for all these cases we had connected the ESBL gene (*bla*_CTX-M-1_) to the correct plasmid (IncI1/ST7) (Tables 1–2). Of the 11 AmpC (*bla*_CMY-2_) genes, ten were connected to their correct plasmid (IncK). The only exception was found again for strain 1047 for which we could not reconstruct an IncK plasmid (Table 2). The above findings show that PLACNET worked efficiently to assemble plasmids from WGS data, although the assignment of small scaffolds to plasmids can be problematic, as is illustrated above by the ESBL genes that were not linked to a specific reconstructed plasmid (see also discussion below and in [23]).

### Identification of distinct ESBL-associated plasmid lineages

Fifteen ESBL genes were connected to a reconstructed plasmid, of which 13 were connected to an IncI1 plasmid. Frequently (eight out of 13), these IncI1 plasmids were also unequivocally linked to other antibiotic resistance genes, such as *sul*, *dfrA*, *aadA* or *tet*. We also found IncK plasmids that were commonly (ten out of 12 plasmids) associated with the AmpC β-lactamase-encoding gene *bla*_CMY-2_ (Fig. 4).

As IncI1 and IncK were the only plasmid families that included reconstructed ESBL-/AmpC-containing plasmids in strains from both humans and animals/meat, we further investigated their potential role in the transfer of resistance genes through the food-chain. To this aim we built a gene content-based dendrogram that also included closely related and publicly available plasmid sequences. In the resulting dendrogram, all reconstructed ESBL-containing IncI1 plasmids, except the *bla*_SHV-12_-carrying plasmids p1A_2 and p9B_1, clustered into one specific branch that did not contain any other previously sequenced plasmid (Fig. 5). This branch also contained 12 of the 13 reconstructed IncI1 plasmids that did not include an ESBL gene. Similarly, all of the reconstructed IncK plasmids, except p87A_5, clustered into one specific branch that did not include any previously sequenced plasmid. These findings suggest the existence of IncI1 and IncK plasmids with a genetic profile distinct from previously characterised plasmids. We did not find any single gene that unequivocally explained the formation of the IncK branch, pointing to a delicate configuration of genes that gives these plasmids their unique genetic profile. However, for the IncI1 branch, we found a characteristic shufflon-related gene (UniProt P10487) that was present in all 26 reconstructed IncI1 plasmids, but which was absent from related IncI1 plasmids (Fig. 5).

To further characterise the IncI1 and IncK resistance plasmids, phylogenetic trees were built from the sequences of the reconstructed plasmids and their closest plasmid relatives. For the IncI1 phylogenetic reconstruction, the 23 plasmids belonging to the specific IncI1 branch as well as 27 related plasmids were included. An OrthoMCL analysis of these plasmids resulted in 8 core proteins (S3 Table), corresponding to a concatenated nucleotide alignment of 8.6 kbp, including 763 variable positions. In the phylogenetic tree built from these variable positions the reconstructed IncI1 plasmids were assigned to four distinct branches (Fig. 6A), each of which also contained previously characterised plasmids. However, the reconstructed plasmids within each branch were always more similar to each other than to any of these previously characterised plasmids. Two of the four branches, corresponding to IncI1/ST3 and IncI1/ST7, contained reconstructed ESBL-harbouring plasmids from both humans and animals or meat. Further rounds of OrthoMCL analyses showed that the reconstructed plasmids within each of these two sets were highly similar to each other: a maximum of only four SNPs (all attributable to p53C_2) was found in the 40 kbp plasmid core of the IncI1/ST3 subset, whereas no SNPs were found in the almost 50 kbp plasmid core of the IncI1/ST7 subset (Fig. 6A). Similarly, a subset of the *bla*_CMY-2_-carrying IncK plasmids contained a plasmid core of almost 37 kbp with a maximum of 27 SNPs (Fig. 6B), which were mostly attributable to p435_1. Leaving out p435_1 from the comparisons revealed a maximum of only seven SNPs. These data strongly support the existence of ESBL-associated IncI1 and AmpC-associated IncK plasmids that have spread through phylogenetically distinct *E. coli* populations, possibly contributing to the dissemination of ESBLs and AmpC-type β-lactamases through the food-chain.

### Validation of plasmid reconstructions by PacBio sequencing of strains 53C and FAP1

To validate the conclusions drawn from the PLACNET reconstructions, we sequenced two strains (53C and FAP1) using long-read DNA sequencing technology (Pacific Biosciences). Strain 53C was selected because it has both an IncI1 and an IncK plasmid, carrying *bla*_CTX-M-1_ and *bla*_CMY-2_, respectively. Strain FAP1 was selected because it contained an IncI1 plasmid of the same lineage as the one in strain 53C (Fig. 6A). The total amount of reconstructed plasmid sequence for strains 53C and FAP1 was 338 kbp and 319 kbp, respectively (Table 2). Genomes were assembled to an average depth of 66.7- and 77.0-fold, respectively, resulting in 11 contigs for strain 53C and five contigs for strain FAP1 (S4 Table). Inspection of the contig sequences showed the presence of four large plasmids in both strains. These were assigned to Inc groups F, I1 (carrying *bla*_CTX-M-1_), I2, and K (carrying *bla*_CMY-2_) in strain 53C and F, I1 (carrying *bla*_CTX-M-1_), and I2, in strain FAP1. A single plasmid in strain FAP1 could not be assigned to an Inc group. Except for the IncI1 plasmid of FAP1, all plasmid contigs could be circularized (S4 Table). The plasmid content was in agreement with our reconstructions, except for two inconsistencies in strain FAP1: (i) PLACNET did not assign a *bla*_CTX-M-1_ gene to its IncI1 plasmid, and (ii) PLACNET reconstructed two IncF plasmids. Blast analysis of both reconstructed IncF plasmids against the FAP1 long-read assembly suggested that they should indeed have been merged into one single plasmid. The reason for this incorrect prediction by PLACNET is unclear, but in the constellation network the two plasmids were relatively far away from each other, suggesting that the IncF plasmid in FAP1 is a fusion between previously observed IncF plasmids present in the reference database. These data show that caution must be taken in case PLACNET predicts multiple plasmids of the same Inc group in one strain. For the remaining plasmids, blast analysis showed that the precision rate of PLACNET was high, ranging from 97–100% (Table 3). Also in terms of sensitivity, PLACNET performed well being able to recover 72.1–99.7% of the plasmids (Table 3). The plasmid regions that were not reconstructed by PLACNET mostly aligned with small scaffolds (average size of 2.0±1.9 kbp, n = 33) in the assemblies built from Illumina short-read data, which indicates that these regions are difficult to assemble. Notably, these small scaffolds encoded many mobile element-, phage-, transposon- and integrase-associated proteins (29.7% of all predicted proteins in these scaffolds) as compared to the correctly assigned scaffolds, where only 6.7% of the proteins had these predicted roles. These observations are in line with results obtained from the PLACNET validation analyses described in [23] and show that PLACNET efficiently reconstructs plasmids from WGS data. Finally, the PLACNET-based prediction that both IncI1 plasmids from strains 53C and FAP1 are highly similar (Fig. 6A) was confirmed by aligning the two complete IncI1 plasmid sequences assembled from the long-read sequencing data. Filtering out repetitively aligning regions resulted in a pairwise alignment of 94.8 kbp containing only 4 SNPs. These data further substantiate our conclusions regarding highly successful plasmid lineages disseminating cephalosporin resistance.

**Table 3:** PLACNET precision and sensitivity rates for seven reconstructed plasmids.

PLACNET plasmid (kbp)	Corresponding PacBio plasmid (kbp)	PLACNET precision (%)	PLACNET sensitivity (%)*
p53C_1 (57.3)	IncI2(56.9)	97.0	96.4
p53C_2 (83.2)	IncI1 (109.7)	99.1	72.1
p53C_3 (69.7)	IncK (86.0)	100	76.7
p53C_4 (128.2)	IncF (134.8)	97.6	90.6
pFAP1_2 (89.7)	IncI1 (129.4)	100	82.1
pFAP1_3 (58.4)	IncI2 (62.4)	100	95.7
pFAP1_5 (46.1)	unclassified plasmid (46.2)	100	99.7

* Note that the region of the PacBio plasmid that was recovered can exceed the reconstructed plasmid size because of repetitive elements, which are collapsed in Illumina assemblies, but are uncollapsed in PacBio assemblies. For each reconstructed plasmid the PLACNET precision rate was calculated by the formula [(assembly size of all correctly assigned scaffolds/assembly size of all correctly+incorrectly assigned scaffolds)×100%]. Sensitivity reflects the percentage of each plasmid sequence (assembled using PacBio data) that was correctly reconstructed in the PLACNET analysis. The sensitivity rate was calculated by the formula [(total nr. of non-overlapping aligning residues/size of the plasmid that was assembled using PacBio data)×100%].

## Discussion

We assessed the epidemiology of ESBL-producing *E. coli* from humans, animals and food using WGS. Our findings strongly suggest the existence of highly successful ESBL-carrying plasmids facilitating transmission of ESBL genes between different reservoirs. This has important implications for our understanding of the dynamics of the spread of ESBL genes and for evaluating control measures.

Several strains that were sequenced in this study and which originated from humans and poultry had previously been considered indistinguishable based on MLST, plasmid and ESBL gene typing, suggesting clonal transfer of these strains through the food-chain, to humans [15]. The claim that ESBL-producing *E. coli* strains from humans and poultry are frequently identical was also made in other studies that made use of traditional sequence-based typing methods [14], [16]. However, as has been demonstrated for different bacterial pathogens and in varied contexts, especially bacterial outbreak investigations, WGS provides superior resolution over traditional typing methods in terms of ruling in and out epidemiological connections between strains [26]–[28]. Similarly, we demonstrate that conclusions on the clonal spread of ESBL-producing *E. coli* through the food-chain cannot realistically be drawn on the basis of traditional sequence-based typing methods, due to their insufficient discriminative power. More specifically, we found that none of the five pairs of human and poultry-associated isolates, previously typed as indistinguishable, were particularly closely related. The most similar pair of isolates differed by 1263 SNPs/Mbp compared to a difference of 1.8 SNPs/Mbp for known/expected clonally related isolates. Hence, inferences from classical typing-based studies regarding the extent of transfer of ESBL-producing *E. coli* strains from animals via food to humans and the burden of disease and mortality due to the use of third-generation cephalosporins in food production must be considered as highly speculative [11].

In fact, our findings strongly suggest that distinct plasmids disproportionately contribute to the spread of antibiotic resistance between different reservoirs. We have demonstrated the existence of highly similar cephalosporin resistance-encoding IncI1/ST3 (40.0 kbp core, 0–4 SNPs), IncI1/ST7 (49.7 kbp core, 0 SNPs), and IncK (36.9 kbp core, 0–27 SNPs) plasmids in different reservoirs. Reconstructed *bla*_CTX-M-1_-carrying IncI1/ST3 plasmids were found in one human and two poultry isolates, *bla*_CTX-M-1_-carrying IncI1/ST7 plasmids were found in three human, two poultry, and one pig isolate; and *bla*_CMY-2_-carrying IncK plasmids were found in five human and four poultry isolates. The isolates carrying these plasmids belonged to evolutionarily distinct backgrounds (IncI1 in phylogroups A, B1 and B2; IncK in phylogroups A, B1, B2, D and F), suggesting that these plasmids efficiently spread through *E. coli* populations and play an important role in the dissemination of ESBL and AmpC-type β-lactamases between different reservoirs.

Based on their genetic content, the IncI1 and IncK plasmids in our dataset clustered into specific sub-branches that did not contain any previously characterised plasmid. However, phylogenetic analyses revealed that these sub-branches could be split into evolutionarily distinct plasmids, some of them being distantly related to previously sequenced plasmids. These findings suggest that evolutionarily distinct plasmids have been accumulating genes from the same genetic reservoir, resulting in plasmids with a similar genetic inventory. The reconstructed IncI1 plasmids all harboured a characteristic shufflon-related gene that was absent from previously characterised IncI1 plasmids. Shufflons are site-specific recombination systems that produce variable C-terminal extensions of the PilV adhesin, resulting in variations of recipient ability in IncI1 plasmid mating [29]. Whether this shufflon explains the promiscuous nature of ESBL-carrying IncI1 plasmids remains to be determined.

One important question is to what extent the IncI1 and IncK resistance plasmids found in this study have spread beyond The Netherlands. Given the trees in Fig. 6, it is clear that currently available plasmid sequences in public databases do not contain any plasmids that are particularly closely related to our reconstructed IncI1 and IncK plasmids. The pMLST repository (http://pubmlst.org/plasmid/) shows that *bla*_CTX-M-1_-carrying IncI1/ST3 plasmids have been isolated from six different European countries, whereas *bla*_CTX-M-1_-carrying IncI1/ST7 plasmids have until now been isolated only from The Netherlands and Germany. The location of *bla*_CMY-2_ on an IncK plasmid, as found here, has only been occasionally reported before, in The Netherlands [30],[31], but also in Sweden [32] and Canada [33]. Future sequencing projects are needed to determine whether the previously identified plasmids isolated outside The Netherlands are closely related to those described here.

We found that none of the human *E. coli* strains in our dataset were closely related to strains from poultry. In contrast, nine out of 17 human isolates (53%) contained a *bla*_CTX-M-1_ or a *bla*_CMY-2_ gene located on plasmids that were highly similar to those found in poultry. These data cannot be interpreted to mean that clonal transfer of antibiotic resistant *E. coli* strains between poultry and humans does not occur, but rather that such transfer occurs less frequently than the transfer of resistance plasmids between both reservoirs. One drawback of our study is that we have used a relatively small sample size (32 strains). Future studies, using larger sample sizes, are needed in order to make more accurate estimates of the relative (and absolute) contributions of clonal versus plasmid transfer towards the spread of antibiotic resistance and the associated health-care burden. In addition, our study focuses on IncI1 and IncK plasmids. Future studies are needed that also focus on other plasmid families, such as IncF plasmids, which are commonly detected in *E. coli* from human infections and are associated with the dissemination of many virulence and antibiotic resistance determinants [34],[35].

Conjugal transfer of plasmids carrying antibiotic resistance genes has been shown to frequently occur among *Enterobacteriaceae* in different environments, including milk, meat, and feces, even in the absence of antibiotic pressure [36],[37]. Moreover, it has been shown that *bla*-carrying plasmids are readily transferred from invading *Enterobacteriaceae* to *Enterobacteriaceae* that are indigenous to the animal and human intestine and that the invading clone itself generally does not persist in the intestine [38], [39]. Nonetheless, it is difficult to infer to what extent the reservoir of *bla*-type resistance genes in poultry contributes to the carriage of such genes by human *E. coli* strains. If successful plasmids are largely responsible for the rising prevalence of ESBL- and AmpC-producing *E. coli* in healthy humans, their emergence in poultry and humans may simply be a reflection of selection of strains carrying these plasmids due to antibiotic usage in human and veterinary medicine.

A better understanding of the dynamics of ESBLs and other resistance genes in different hosts is needed to design effective control measures, both in the community and within health care settings. Our findings strongly suggest the occurrence of clonal transfer of ESBL-producing *E. coli* between pigs and pig farmers, which may well occur through direct contact or through aerosols. Whether such events represent a public health threat remains to be determined. The occurrence of transmission of ESBL-producing *E. coli* from poultry through the food-chain is less evident. The occurrence of highly-related plasmids that carry ESBL- and AmpC-type resistance genes among genotypically distinct *E. coli* strains from different sources is cause for concern because this suggests that plasmids can spread with relative ease between the different reservoirs and the spread of these plasmids may be exceedingly difficult to control. Clearly, there still remains an urgent need to minimize the use of third-generation cephalosporins in animal husbandry as this is an important selective pressure for the occurrence of ESBL- and AmpC-producing *E. coli* in animals raised for food production.

## Materials and Methods

### Isolates and molecular analyses

The genomes of 32, mostly ESBL-producing, *E. coli* strains isolated from different reservoirs in The Netherlands in the period 2006–2011, were sequenced. One set of isolates (n = 24) has been studied previously using classical typing methods [15], [18]. This set contained strains from human clinical infections (n = 13) which had been obtained from geographically dispersed laboratories in The Netherlands, servicing secondary and tertiary care hospitals, general practitioners and long-term care facilities. Additional isolates were from chickens raised on production farms (n = 4) and chicken retail meat (n = 7) (Table 1). All 24 isolates were previously genotyped by MLST [40] (http://mlst.warwick.ac.uk/mlst/dbs/Ecoli) and plasmid characterization was previously performed using PCR-based replicon typing [31], [41] and additional pMLST for IncI1 plasmids [42], [43] (http://pubmlst.org/plasmid/). Detection of ESBL genes had been performed for all 24 strains using microarray analysis and gene sequencing [44]. In addition, detection of AmpC-type β-lactamase-encoding genes had been performed for 11 strains, using gene sequencing [18]. The association between ESBL/AmpC genes and plasmids was previously determined by both Southern blot hybridization and transformation [31]. Four non-ESBL-producing isolates were included as controls and were analysed for the carriage of plasmids that can incorporate ESBL genes via horizontal gene transfer. The second set of isolates contained eight ESBL-producing strains that had been isolated from three different pig farms in The Netherlands in 2011 (Table 1). These farm strains were part of a larger cohort that will be described in detail elsewhere (Dohmen *et al.*, unpublished data). For one farm (farm A), four strains were collected, two from different fecal pools of six unique pigs and two from the feces of different farmers. For each of the other two farms (farms B and C), one strain was collected from a fecal pool of six pigs and one from the feces of a farmer. Detection of the ESBL (*bla*_CTX-M-1_) gene was performed using a CTX-M-1 group-specific PCR and additional gene sequencing (Dohmen *et al.*, unpublished data).

### Genome sequencing and assembly

Genomic DNA was isolated from cell pellets using the Ultraclean Microbial DNA isolation kit (Mo Bio Laboratories, Inc., Carlsbad, CA, USA) according to the manufacturer's instructions. Strains were sequenced using Illumina HiSeq 2000 sequencing technology (Illumina, Inc., San Diego, CA, USA) generating 90 bp paired-end reads from a library with an average insert size of 500 bp and a total amount of quality-filtered raw sequence of over 600 Mbp per strain. Quality filtering included the removal of duplicate reads and reads that contained ≥15 bp overlap with the adapter sequences. The corresponding paired-end reads were also removed in these cases. Reads were assembled *de novo* using SOAPdenovo v1.05 [45]. For each Illumina dataset, a range of different k-mer lengths (21–63 bp) was empirically tested to obtain the assembly with the lowest number of scaffolds of size ≥500 bp. In cases where more than one assembly contained the lowest number of scaffolds, the parameters of choice to pick the best assembly were: the lowest number of contigs of size ≥200 bp, the highest N50 for the scaffolds, and the highest N50 for the contigs, in order of priority. Assembly statistics are reported in S1 Table. Two strains (53C and FAP1, Table 1) were also sequenced on a Pacific Biosciences RS II instrument (Pacific Biosciences, Inc., Menlo Park, CA, USA). Libraries were prepared using the PacBio 20 kbp library preparation protocol. Size selection (5 kbp cut-off) of the final libraries was performed using a BluePippin instrument (Sage Science, Inc., Beverly, MA, USA). Sequencing was performed using P4-C2 chemistry. Three and five SMRT cells were used for sequencing strains FAP1 and 53C, respectively, generating 159191 and 95263 reads and a total of 997.1 and 471.5 Mbp, respectively. Reads were assembled using HGAP v3 (Pacific Biosciences, SMRT Analysis Software v2.2.0). Minimus2, part of the AMOS package [46], was used to circularize contigs. The SMRT Analysis Software was used to map reads back to the contigs and correct sequences after circularization. Assembly statistics are reported in S4 Table.

### Sequence data analysis

Publicly available sequence data were retrieved from GenBank (ftp://ftp.ncbi.nih.gov/genomes/Bacteria and ftp://ftp.ncbi.nih.gov/genomes/Bacteria_DRAFT). Whole genome sequence data for 126 *Escherichia* and 12 *Shigella* species were downloaded in June 2012, whereas sequence data for 4188 completely sequenced plasmids, 797 of them from *Enterobacteriaceae*, were downloaded in June 2013. The strains that were sequenced in this study were annotated with RAST v4.0 [47] using default settings. Predicted proteins were assigned to Clusters of Orthologous Groups (COG) [48] as described previously [49]. On the basis of COG assignments, a core proteome was defined by (i) extracting, per analysed genome, all proteins with one or more COGs assignments and which represented the only protein in that given COG or combination of COGs and by (ii) selecting from those proteins the ones that occurred in all genomes analysed. Alternatively, in the cases where smaller genomic datasets were analysed (see Results), core proteomes were determined by first subjecting all associated protein sequences to an all-vs-all blastp similarity search (defaults settings, except for: -F ‘m S’; -e 1×10^−5^; -z [the total number of proteins used in the analysis]). Groups of orthologous proteins were determined from the blastp output using OrthoMCL v2.0.2 [20]. Orthologous groups with exactly one representative protein from each input genome were considered to be part of the core proteome. Core genome alignments were built as follows: for each group of orthologous proteins, the corresponding nucleotide sequences were extracted and aligned using Muscle v3.7 [50], after which gaps were stripped from each alignment using trimAl v1.2 [51]. The resulting alignments were concatenated to yield a core genome alignment. Phylogenies were reconstructed by building maximum likelihood phylogenetic trees from the variable positions in core genome alignments using RAxML v7.2.8 [52] under the GTRCAT model. Confidence was inferred by running 100 or 1000 bootstrap replicates under the same model. Trees were mid-point rooted and visualised in MEGA v5.05 [53]. Bowtie2 [54] was used for mapping Illumina reads against scaffolded assemblies and gene sequences. MLST profiling of sequenced bacteria was performed using MLST v1.6 [55]. Pairwise large-scale nucleotide alignments were built using NUCmer v3.23 (with –mum option), which is part of the MUMmer package [56].

### Plasmid reconstructions from WGS data

Plasmid reconstructions were based on the Plasmid Constellation Networks (PLACNET) method of genome representation [23]. In short, for all genomes, a PLACNET representation that clusters all plasmid-associated contigs was built using (i) contig similarities with reference genomes, (ii) all possible contig linkages, and (iii) plasmid-specific relaxase and replication initiator genes. This information was implemented in a network, where genomic contigs, together with reference plasmid and genome sequences are shown as nodes. The nodes are linked by edges of homology and scaffolding information. As a result, contigs fall into clusters, the largest one being the chromosome and additional ones being plasmids. Manual curation of the resulting networks helped solving most of the remaining ambiguities. Reference data from GenBank contained 4188 plasmids and 2728 chromosomes. Contig similarity analysis was performed using megablast against these reference data. Contig homology edges were defined by the five best blast hits (e-value <1×10^−20^). Scaffolds were determined by mapping all reads against contigs using Bowtie2 [54], and allocating as scaffold links all discordant paired-end reads that matched two different contigs. To provide additional evidence for the plasmid origin of a cluster, a blastp search against in-house databases containing plasmid-specific relaxases and replication initiator proteins was performed. Contigs encoding these proteins were tagged in the PLACNET. Plasmid Neighbour-Joining dendrograms were built based on previously described methodologies [57] using CD-HIT [58] to construct protein profiles and the Jaccard formula to calculate distance metrics between profiles. PLACNET results were validated as follows: for the two strains 53C and FAP1, the scaffolds assigned to the reconstructed plasmids were queried using megablast against the assemblies resulting from Pacific Biosciences (PacBio) sequencing. The best blast hit (e-value ≤1×10^−10^) was inspected to assess whether the scaffolds had been assigned to the correct plasmid. For each reconstructed plasmid the PLACNET precision rate was calculated by the formula [(assembly size of all correctly assigned scaffolds/assembly size of all correctly+incorrectly assigned scaffolds)×100%]. To assess PLACNET sensitivity (the percentage of each plasmid sequence size that was recovered) blast hits against the corresponding PacBio plasmids were collected (e-value ≤1×10^−10^, minimum of 250 identical residues). The sensitivity rate was calculated by the formula [(total nr. of non-overlapping aligning residues found by blast/size of the PacBio plasmid)×100%].

### Accession numbers

All sequence data have been deposited at DDBJ/EMBL/GenBank. Accession numbers for the Illumina sequence data are listed in Table 1. Pacific Biosciences sequence data have been deposited with accession numbers PRJNA260957 for strain 53C and PRJNA260958 for strain FAP1.

## Supporting Information

S1 Table Assembly statistics of genome sequences of strains sequenced with Illumina technology.
(DOCX)

S2 Table Assembled *bla*_TEM_ genes and ambiguous nucleotide positions.
(DOCX)

S3 Table Core proteomes of IncI1 and IncK plasmids.
(DOCX)

S4 Table Assembly statistics of genome sequences of strains 53C and FAP1 (sequenced with Pacific Biosciences long-read technology).
(DOCX)
