# Supplementary material for: Dissemination of Cephalosporin Resistance Genes between Escherichia coli Strains from Farm Animals and Humans by Specific Plasmid Lineages
Source: PLoS Genet. 2014 Dec 18;10(12):e1004776. doi: 10.1371/journal.pgen.1004776 (PMC4270446; doi:10.1371/journal.pgen.1004776)
Supplement: S1 Table — Assembly statistics of genome sequences of strains sequenced with Illumina technology. (DOCX) [file pgen.1004776.s001.docx]

**Table S1. Assembly statistics of genome sequences of strains sequenced with Illumina technology.**

| **Strain** | **# scaffolds** | **N50 (bp)** | **N90 (bp)** | **Longest scaffold (bp)** | **Average Nt coverage** | **GC%** | **Total assembled sequence (Mbp)** | **# genes** |
| --- | --- | --- | --- | --- | --- | --- | --- | --- |
| 148 | 112 | 125332 | 30827 | 296899 | 120 | 50.57 | 4.91 | 4767 |
| 320 | 103 | 147419 | 37926 | 367231 | 117 | 50.69 | 5.11 | 4964 |
| 681 | 96 | 159817 | 42261 | 396973 | 117 | 50.73 | 5.08 | 4932 |
| 38.27 | 182 | 95667 | 18156 | 323445 | 112 | 50.29 | 5.20 | 5073 |
| 38.34 | 179 | 109891 | 19368 | 452240 | 110 | 50.30 | 5.33 | 5246 |
| 53A | 89 | 197763 | 37922 | 833818 | 122 | 50.65 | 4.88 | 4805 |
| 85B | 113 | 144724 | 37770 | 410503 | 116 | 50.34 | 5.11 | 5020 |
| 1240 | 147 | 194347 | 27095 | 631572 | 112 | 50.62 | 5.26 | 5024 |
| 1350 | 153 | 109054 | 28271 | 564784 | 113 | 50.59 | 5.21 | 5147 |
| 1365 | 112 | 135615 | 30837 | 475403 | 117 | 50.68 | 5.09 | 4947 |
| 38.16 | 106 | 176995 | 30927 | 331015 | 119 | 50.66 | 5.00 | 4830 |
| 897 | 130 | 217264 | 40739 | 601538 | 113 | 50.69 | 5.23 | 5083 |
| 1047 | 114 | 226645 | 32629 | 579834 | 115 | 50.73 | 5.14 | 4983 |
| 38.52 | 154 | 147220 | 19997 | 396795 | 113 | 50.57 | 5.20 | 5017 |
| 53C | 138 | 158467 | 26710 | 464801 | 109 | 50.62 | 5.41 | 5276 |
| 435 | 184 | 160556 | 26937 | 485993 | 106 | 50.42 | 5.44 | 5230 |
| 328 | 126 | 136750 | 30766 | 456828 | 109 | 50.62 | 5.45 | 5335 |
| 597 | 62 | 318266 | 46813 | 536123 | 119 | 50.57 | 5.02 | 4924 |
| 668 | 111 | 198351 | 24899 | 511643 | 115 | 50.58 | 5.17 | 5012 |
| 606 | 151 | 119134 | 25096 | 391442 | 110 | 50.62 | 5.34 | 5187 |
| 1A | 95 | 202867 | 31244 | 448803 | 115 | 50.54 | 5.11 | 4971 |
| 27A | 132 | 175774 | 23021 | 411303 | 114 | 50.49 | 5.13 | 4983 |
| 9B | 249 | 106534 | 14198 | 257148 | 107 | 50.61 | 5.41 | 5326 |
| 87A | 242 | 99611 | 13081 | 209419 | 100 | 50.38 | 5.40 | 5041 |
| FAH1 | 143 | 154416 | 30162 | 371825 | 114 | 50.67 | 5.13 | 5177 |
| FAH2 | 98 | 155406 | 37441 | 318151 | 117 | 50.62 | 5.05 | 5029 |
| FAP1 | 112 | 155398 | 33965 | 371715 | 116 | 50.46 | 5.12 | 5116 |
| FAP2 | 106 | 155350 | 37417 | 371666 | 117 | 50.51 | 5.07 | 5067 |
| FBH1 | 114 | 103452 | 23824 | 344819 | 118 | 50.73 | 5.00 | 4941 |
| FBP1 | 139 | 100668 | 30761 | 286798 | 116 | 50.55 | 5.10 | 5152 |
| FCH1 | 162 | 91851 | 27790 | 217493 | 116 | 50.66 | 5.06 | 5092 |
| FCP1 | 98 | 118819 | 25417 | 297448 | 125 | 50.59 | 4.73 | 4641 |

All statistics shown are based on the scaffolded assemblies and scaffolds of size ≥ 500 bp.
