# Supplementary material for: Dissemination of Cephalosporin Resistance Genes between Escherichia coli Strains from Farm Animals and Humans by Specific Plasmid Lineages
Source: PLoS Genet. 2014 Dec 18;10(12):e1004776. doi: 10.1371/journal.pgen.1004776 (PMC4270446; doi:10.1371/journal.pgen.1004776)
Supplement: S2 Table — Assembled bla TEM genes and ambiguous nucleotide positions. (DOCX) [file pgen.1004776.s002.docx]

**Table S2. Assembled *bla*_TEM_ genes and ambiguous nucleotide positions.**

| **Strain** | **Typed *bla*_TEM_ gene (screening for *bla*_TEM-1_ was not performed)** | ***bla*_TEM_ gene in WGS** | **Protein length** | ***bla*_TEM_ average Nt coverage ± s.d.** | ***bla*_TEM_ relative coverage*** | **Position of ambiguous Nt (& corresp. aa position)†** | **Coverage of ambiguous Nt** | **Most abundant Nts at ambiguous position (& resulting aa). Consensus (assembled) Nts are in bold.** |
| --- | --- | --- | --- | --- | --- | --- | --- | --- |
| 320 | TEM-52 | TEM-20 | 280 aa | 325.0 ± 79.4 | 2.8 | 310 (104) | 337 | 175 × A (Lys), **162 × G (Glu)** |
|  |  |  |  |  |  | 402 (134) | 437 | **232 × G (Ala)**, 205 × T (Ala) |
|  |  |  |  |  |  | 545 (182) | 347 | **192 × C (Thr)**, 155 × T (Met) |
|  |  |  |  |  |  | 712 (238) | 233 | **120 × A (Ser)**, 113 × G (Gly) |
| 681 | TEM-52 | TEM-20 | 280 aa | 295.4 ± 70.8 | 2.5 | 310 (104) | 295 | 151 × A (Lys), **144 × G (Glu)** |
|  |  |  |  |  |  | 402 (134) | 403 | **222 × G (Ala)**, 181 × T (Ala) |
|  |  |  |  |  |  | 545 (182) | 323 | **179 × C (Thr)**, 142 × T (Met) |
|  |  |  |  |  |  | 712 (238) | 225 | **119 × A (Ser)**, 106 × G (Gly) |
| 38.34 | TEM-52 | TEM-1 | 280 aa | 326.4 ± 73.9 | 3.0 | 310 (104) | 311 | **168 × G (Glu)**, 143 × A (Lys) |
|  |  |  |  |  |  | 402 (134) | 399 | **215 × T (Ala)**, 184 × G (Ala) |
|  |  |  |  |  |  | 545 (182) | 426 | **228 × T (Met)**, 198 × C (Thr) |
|  |  |  |  |  |  | 712 (238) | 240 | **120 × G (Gly)**, 116 × A (Ser) |
| 85B | TEM-52 | TEM-52 | 286 aa | 181.2 ± 36.7 | 1.6 | - | - | - |
| 27A | TEM-52 | TEM-52 | 286 aa | 125.9 ± 23.8 | 1.1 | - | - | - |
| 148 | - | TEM-1 | 286 aa | 171.0 ± 24.0 | 1.4 | - | - | - |
| 1240 | - | TEM-1 | 286 aa | 413.0 ± 75.2 | 3.7 | 545 (182) | 407 | **221 × T (Met)**, 186 × C (Thr) |
| 1350 | - | TEM-1 | 286 aa | 169.1 ± 26.5 | 1.5 | - | - | - |
| 1365 | - | TEM-1 | 286 aa | 170.6 ± 28.0 | 1.5 | - | - | - |
| 38.16 | - | TEM-1 | 286 aa | 315.7 ± 38.3 | 2.7 | - | - | - |
| 328 | - | TEM-1 | 286 aa | 174.1 ± 22.3 | 1.6 | - | - | - |
| 668 | - | TEM-1 | 286 aa | 106.6 ± 19.5 | 0.9 | - | - | - |
| 606 | - | TEM-1 | 286 aa | 120.4 ± 16.3 | 1.1 | - | - | - |
| 87A | - | TEM-1 | 286 aa | 194.7 ± 37.6 | 1.9 | - | - | - |
| FAH1 | - | TEM-1 | 286 aa | 142.6 ± 21.1 | 1.3 | - | - | - |
| FAH2 | - | TEM-1 | 286 aa | 153.9 ± 22.0 | 1.3 | - | - | - |
| FAP1 | - | TEM-1 | 286 aa | 155.0 ± 21.1 | 1.3 | - | - | - |
| FAP2 | - | TEM-1 | 286 aa | 143.9 ± 26.3 | 1.2 | - | - | - |

* *bla*_TEM_ relative coverage was calculated by dividing the average *bla*_TEM_ coverage by the average genomic coverage (Table S1).

**†** Amino acid positions are in correspondence with the amino acid positions listed for TEM-1 by the Lahey clinic (www.lahey.org/Studies/temtable.asp).

Ambiguous positions were found as follows: first, the raw Illumina reads of each strain were mapped against its own assembly (allowing a maximum of one mismatch per seed). Second, positions were designated as ambiguous when at least 50 reads mapped to it and when the second most abundantly mapped residue had a share of ≥ 25% of all mapped residues at that position. Positions covered by indels were not taken into account. The four ambiguous positions found in the assembled *bla*_TEM_ genes of strains 320, 681 and 38.34 exactly corresponded to the four SNPs that differentiate *bla*_TEM-1_ and *bla*_TEM-52_ in the same genetic region. This suggested that both *bla*_TEM-1_ and *bla*_TEM-52_ are present in these strains. In strains 320 and 681 this appears to have resulted in an erroneous *bla*_TEM-20_ assembly (i.e. a hybrid between *bla*_TEM-1_ and *bla*_TEM-52_). The single ambiguous position found in the assembled *bla*_TEM_ gene of strain 1240 may point to the presence of *bla*_TEM-1_ and *bla*_TEM-135_ in this strain.
