# Supplementary material for: Dissemination of Cephalosporin Resistance Genes between Escherichia coli Strains from Farm Animals and Humans by Specific Plasmid Lineages
Source: PLoS Genet. 2014 Dec 18;10(12):e1004776. doi: 10.1371/journal.pgen.1004776 (PMC4270446; doi:10.1371/journal.pgen.1004776)
Supplement: S3 Table — Core proteomes of IncI1 and IncK plasmids. (DOCX) [file pgen.1004776.s003.docx]

**Table S3 Core proteomes of IncI1 and IncK plasmids.**

| **Inc group (nr of plasmids analysed)** | **Core protein locus tags of representative plasmids (R64 for IncI1, pCT for IncK)** | **Annotation** | **Protein name** |
| --- | --- | --- | --- |
| IncI1 (50) | R64_p081 | relaxase | NikB |
|  | R64_p090 | conjugal transfer protein | TraX |
|  | R64_p123 | type IV prepilin | PilS |
|  | R64_p124 | integral membrane protein | PilR |
|  | R64_p125 | type IV pilus ATPase | PilQ |
|  | R64_p126 | type IV pilus protein | PilP |
|  | R64_p127 | type IV pilus protein | PilO |
|  | R64_p129 | type IV pilus protein | PilM |
| IncK (17) | pCT_053 | conjugal transfer protein | TrbC |
|  | pCT_054 | conjugal transfer protein | TrbB |
|  | pCT_057 | endonuclease | ParB |
|  | pCT_064 | conserved hypothetical plasmid protein | - |
|  | pCT_068 | conjugal transfer integral membrane protein | TraY |
|  | pCT_069 | conjugal transfer protein | TraX |
|  | pCT_070 | conjugal transfer protein | TraW |
|  | pCT_071 | conjugal transfer protein | TraV |
|  | pCT_072 | conjugal transfer nucleotide-binding protein | TraU |
|  | pCT_073 | conjugal transfer protein | TraT |
|  | pCT_074 | conjugal transfer protein | TraS |
|  | pCT_075 | conjugal transfer protein | TraR |
|  | pCT_076 | conjugal transfer protein | TraQ |
|  | pCT_077 | conjugal transfer protein | TraP |
|  | pCT_078 | conjugal transfer protein | TraO |
|  | pCT_079 | conjugal transfer protein | TraN |
|  | pCT_080 | conjugal transfer protein | TraM |
|  | pCT_087 | conjugal transfer protein | TraK |
|  | pCT_088 | conjugal transfer ATPase protein | TraJ |
|  | pCT_089 | conjugal transfer lipoprotein | TraI |
|  | pCT_090 | conjugal transfer protein | TraH |
|  | pCT_092 | conjugal transfer protein | TraE |
|  | pCT_107 | type IV pilus protein | PilP |
|  | pCT_109 | type IV pilus outer membrane protein | PilN |
|  | pCT_110 | type IV pilus protein | PilM |
|  | pCT_111 | type IV pilus lipoprotein | PilL |
|  | pCT_113 | type IV pilus protein | PilI |
