# Supplementary material for: Dissemination of Cephalosporin Resistance Genes between Escherichia coli Strains from Farm Animals and Humans by Specific Plasmid Lineages
Source: PLoS Genet. 2014 Dec 18;10(12):e1004776. doi: 10.1371/journal.pgen.1004776 (PMC4270446; doi:10.1371/journal.pgen.1004776)
Supplement: S4 Table — Assembly statistics of genome sequences of strains 53C and FAP1 (sequenced with Pacific Biosciences long-read technology). (DOCX) [file pgen.1004776.s004.docx]

**Table S4. Assembly statistics of genome sequences of strains 53C and FAP1 (sequenced with Pacific Biosciences long-read technology).**

| **Strain** | **Contig** | **Circular?** | **Molecule type** | **Length (kbp)** | **Average Nt coverage** | **GC%** | **# genes** |
| --- | --- | --- | --- | --- | --- | --- | --- |
| 53C | 1 | No | Chromosome | 2271.2 | 70 | 51.20 | 2266 |
|  | 2 | No | Chromosome | 1367.7 | 58 | 50.38 | 1416 |
|  | 3 | No | Chromosome | 880.1 | 71 | 50.78 | 890 |
|  | 4 | No | Chromosome | 637.0 | 57 | 50.49 | 669 |
|  | 5 | Yes | IncF plasmid | 134.8 | 88 | 49.53 | 143 |
|  | 6 | Yes | IncI1 plasmid | 109.7 | 78 | 50.95 | 124 |
|  | 7 | Yes | IncK plasmid | 86.0 | 105 | 52.67 | 106 |
|  | 8 | Yes | IncI2 plasmid | 56.9 | 79 | 42.27 | 77 |
|  | 9 | No | Phage | 18.7 | 20 | 55.19 | 40 |
|  | 10 | No | ? | 1.5 | 6.7 | 32.27 | 0 |
|  | 11 | No | ? | 0.9 | 2.7 | 31.77 | 1 |
| FAP1 | 1 | No | Chromosome | 4874.7 | 140 | 50.71 | 4855 |
|  | 2 | Yes | IncF plasmid | 141.8 | 185 | 50.49 | 155 |
|  | 3 | No | IncI1 plasmid | 129.4 | 219 | 50.07 | 143 |
|  | 4 | Yes | IncI2 plasmid | 62.4 | 112 | 42.32 | 81 |
|  | 5 | Yes | plasmid | 46.2 | 90 | 44.24 | 62 |
